# Commentary: Malaria elimination in India and regional implications

**DOI:** 10.3389/fmicb.2018.00992

**Published:** 2018-05-15

**Authors:** Kwang Seung Park, Sumera Kausar Malik, Jung Hee Lee, Asad Mustafa Karim, Sang Hee Lee

**Affiliations:** ^1^National Leading Research Laboratory of Drug Resistance Proteomics, Department of Biological Sciences, Myongji University, Yongin, South Korea; ^2^Department of Bioinformatics, Quaid-i-Azam University Islamabad, Islamabad, Pakistan

**Keywords:** malaria, prevalence, India, bordering countries, *Plasmodium vivax*, *Plasmodium falciparum*, drug resistance

Malaria has remained a greatest health and socioeconomic burden in the tropical and subtropical regions of the world. According to World Health Organization, approximately, 429,000 deaths with 212 million cases were reported from malaria in the world (World Health Organization, 2016a).

Wangdi et al. (2016) in their recent review in “Lancet Infectious Diseases” reported nationwide malaria elimination efforts and challenges in India and bordering countries as India contributes a substantial burden of malaria outside sub-Saharan Africa, with the third highest *Plasmodium vivax* prevalence in the world. Authors have discussed that India shares long international borders with Nepal, China, and Bhutan in the north, Bangladesh and Myanmar in the east, and Pakistan in the west. We agree that malaria remains a substantial public health problem in India and these bordering countries and a cross-border strategy and collaboration with these countries is vital to achieve the goals of malaria elimination in the region. Nevertheless, we felt it was important to draw attention and highlight other potentially significant aspects overlooked by this review according to our (Karim et al., 2016) and other reports (Directorate of Malaria Control Pakistan, 2015; World Health Organization, 2016a).

To highlight that the cross-country collaboration to control malaria is needed, we searched the prevalence (%) of *Plasmodium* species in bordering countries of India. In additon, Ahghanistan was included in bordering countries of India because India shares longest land border with Bangladesh (4,096.7 Km), China (3,488 Km), and Pakistan (3,323 Km) and it shares the shortest land border with Afghanistan (106 Km) (Ministry of Home Affairs, Department of Border Management, 2018). China, Pakistan, and Afghanistan are the most populous countries affected by *Plasmodium vivax* malaria (World Health Organization, 2016b). Our epidemiological and clinical study of bordering region in Pakistan showed that *P. vivax* was the most prevalent species in the area especially alongside regions bordering the neighboring Afghanistan, exhibiting same genetic background (Karim et al., 2016). According to World Health Organization, the prevalence of *P. vivax* in Afghanistan is the highest (95%) among all the bordering countries of India (World Health Organization, 2016b). The prevalence of *P. vivax* in the other bordering countries of India is highest in Pakistan (81%), China (79%), Nepal (78%), and Bhutan (60%) while its prevalence in Myanmar and Bangladesh is 34 and 7%, respectively (Figure 1) (World Health Organization, 2016b). However, *Plasmodium falciparum* is predominant in Bangladesh (93%) followed by Myanmar (66%), Bhutan (40%), Nepal (22%), Pakistan (19%), China (11%), and Afghanistan (5%) (Figure 1). These results suggest that the high prevalence of *P. falciparum* in Bangladesh and Myanmar is likely to contribute to its prevalence (67%) in the northeast region of India (World Health Organization, 2016b). In addition, the high prevalence of *P. vivax* in Afghanistan, Pakistan, China, Nepal, and Bhutan is also likely to contribute to its incidence in the west and north regions of India (World Health Organization, 2016b). Therefore, the cross-country collaboration to control malaria is urgently needed.

**Figure 1:**
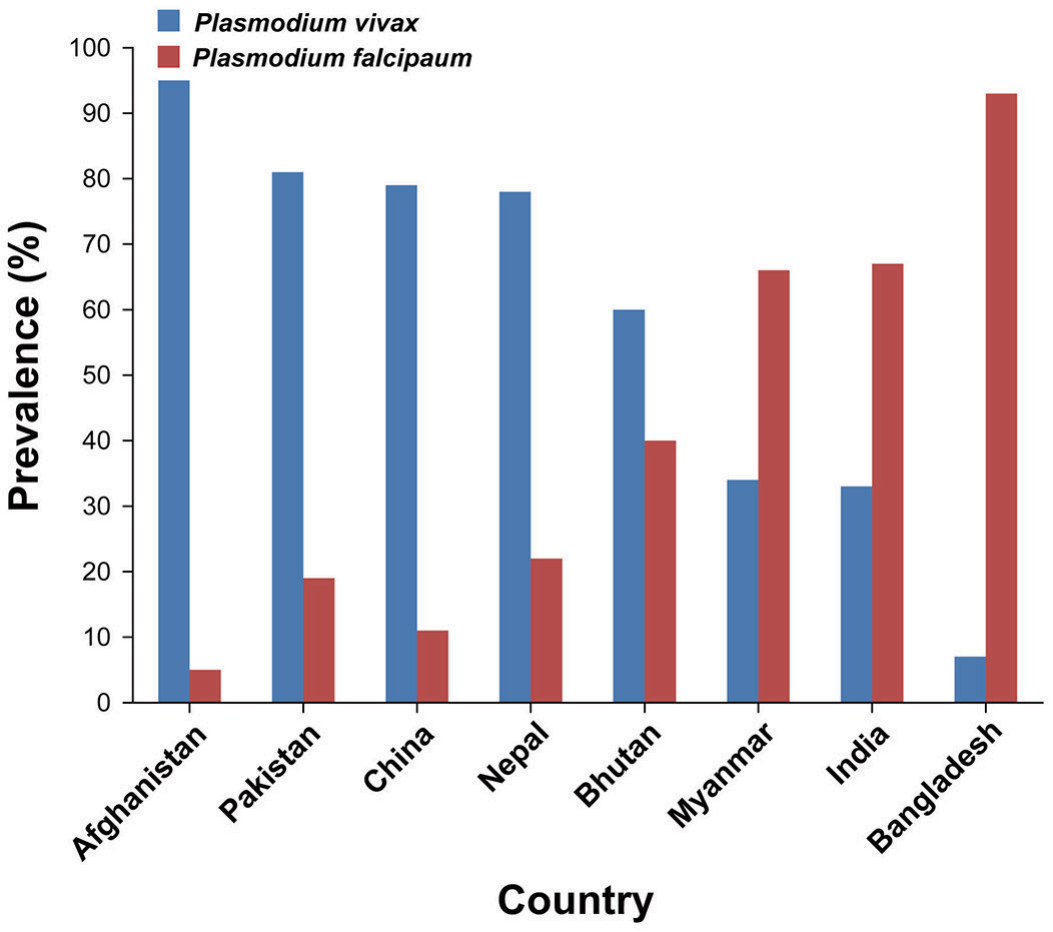
Prevalence (%) of *Plasmodium* species in India and its bordering countries. According to recent reports (World Health Organization, 2016b; Mercado et al., 2017), the numbers of confirmed malaria cases caused by *Plasmodium vivax* in India and its bordering countries were as follows: Afghanistan (*n* = 98,208), Pakistan (*n* = 163,631), China (*n* = 31), Nepal (*n* = 505), Bhutan (*n* = 62), Myanmar (*n* = 62,141), India (*n* = 385,856), and Bangladesh (*n* = 2,772). The numbers of confirmed malaria cases caused by *Plasmodium falciparum* in India and its bordering countries were as follows: Afghanistan (*n* = 5,169), Pakistan (*n* = 38,382), China (*n* = 4), Nepal (*n* = 142), Bhutan (*n* = 42), Myanmar (*n* = 120,626), India (*n* = 783,405), and Bangladesh (*n* = 36,828).

*P. falciparum* was resistant against chloroquine throughout India but sensitive to mefloquine as echoed by Wangdi et al. (2016). However, in all seven bordering countries, extensive chloroquine drug resistance has been emerged in a high proportion (Centers for Disease Control and Prevention, 2017). According to a study, chloroquine resistance is associated with point mutations in the *pfcrt* (*P. falciparum* chloroquine resistance transporter) gene in this fatal human malaria parasite (Pulcini et al., 2015). Additionally, *Plasmodium* species from Myanmar and China are resistant to chloroquine as well as mefloquine (Centers for Disease Control and Prevention, 2017). Resistance in *P. falciparum* to mefloquine is associated with increased expression of *pfmdr1* (*P. falciparum* multidrug-resistant gene 1) gene (Preechapornkul et al., 2009). As the case of chloroquine resistance, there can be mefloquine-resistant *Plasmodium* species exchange between India and the bordering countries. It is more probable that higher antimalarial chloroquine and mefloquine resistance in neighboring Myanmar, Bangladesh, and Pakistan can contribute to increasing resistance in India. (Ghanchi et al., 2011; Wangdi et al., 2016). Previous studies in Southeast Asia have shown that *P. vivax* isolates with *pvmdr1* (*P. vivax* multidrug-resistant gene 1) gene amplification were characterized by increased susceptibility to chloroquine but decreased susceptibility to mefloquine (Imwong et al., 2008; Suwanarusk et al., 2008). Nevertheless, the role of *pvmdr1* in conferring resistance to chloroquine is still elusive and controversial (Faway et al., 2016). Although number of *pvmdr1* gene copies correlated with mefloquine use history, *in vivo* data showing a direct relationship between mefloquine resistance and *pvmdr1* gene amplification form mefloquine-treated patients are needed (Khim et al., 2014). Therefore, further studies are needed to identify trustworthy gene(s) for chloroquine and mefloquine resistance in *P. vivax*.

Kinley Wangdi and colleagues have highlighted the malaria elimination efforts carried out by the neighboring countries, however, they did not mention the Malaria Control Programme of Pakistan bordering Gujarat and Rajasthan, the high malaria transmission western states of India (Wangdi et al., 2016). In 2015, the Government of Pakistan ensured the reinforcement of surveillance and assessment programmes in the country (Directorate of Malaria Control Pakistan, 2015). According to this malaria strategic plan (by 2020), the main goal is to reduce malaria burden by 75% in high and moderate endemic areas and eliminate malaria from low endemic areas of Pakistan, in collaboration with Global Malaria Plan of Action and Global Technical Strategy 2015-20 (Directorate of Malaria Control Pakistan, 2015). Pakistan has made substantial progress in reducing malaria after the implementation of program. The country has achieved lowest annual parasite incidence in Punjab and Azad Kashmir provinces (Directorate of Malaria Control Pakistan, 2016). In 2016, Pakistan was able to make considerable progress in terms of enhanced coverage of malaria control interventions in endemic districts including malaria case management, distribution of Long Lasting Insecticidal Nets (LLINs) and Behavior Change Communication (BCC) activities. As compared to year 2015 (no of LLINs = 1.74 million), 2.4 million LLINs were distributed in the country during 2016. Therefore, these malaria control interventions will help to reduce infection in the future (Directorate of Malaria Control Pakistan, 2016). Therefore, a cross-border malaria elimination strategy between India and Pakistan is urgently needed.

Wangdi and colleagues have overlooked the malaria deaths and prevalence in children. According to WHO, “A child dies of malaria every 2 minutes” (World Health Organization, 2016a). Of 429,000 deaths, 303,000 malaria deaths occurred in children aged under 5 years (World Health Organization, 2016a). Malaria is one of the world's top killer diseases for the young children. According to our study, children are more susceptible to malarial parasite because they have not yet developed immunity against malaria (Karim et al., 2016). Therefore, efforts aimed at the prevention of malaria in young children must be multifaceted. We emphasize that insect avoidance is critical in children and it can prevent the malaria acquisition in them. Rapid diagnosis and treatment of malaria infection are very useful to limit morbidity and mortality caused by it. Cross-border collaboration and global and regional efforts to combat malaria are very critical to achieve the goals of its elimination in the region.

## Author contributions

All authors listed have made a substantial, direct and intellectual contribution to the work, and approved it for publication.

### Conflict of interest statement

The authors declare that the research was conducted in the absence of any commercial or financial relationships that could be construed as a potential conflict of interest.
